# Classroom Interventions and Foreign Language Anxiety: A Systematic Review With Narrative Approach

**DOI:** 10.3389/fpsyg.2021.614184

**Published:** 2021-02-09

**Authors:** Michiko Toyama, Yoshitaka Yamazaki

**Affiliations:** Faculty of Business Administration, Bunkyo University, Tokyo, Japan

**Keywords:** anxiety reduction, experimental studies, educational intervention, learning environment, foreign language classroom anxiety, interactions

## Abstract

Experimental studies have developed, conducted, and evaluated classroom interventions for foreign language anxiety (FLA) reduction. However, various characteristics of those classroom interventions make it difficult to synthesize the findings and apply them to practice. We conducted what is, to the best of our knowledge, the first systematic review on educational interventions for FLA. Six criteria were established for inclusion of studies. Using English keywords, we identified 854 potentially eligible studies through ProQuest and Scopus, 40 of which were finally included. All included studies were published from 2007 to 2020. The studies differed in type of intervention, duration of intervention, and scale to measure FLA. Our systematic review resulted in seven features of classroom interventions, categorized as student–student interactions, student-teacher interactions, self-management, and mood boosters; we also categorized interventions as either individual or interactional.

## Introduction

A second language, which is often referred to as L2, is a language that is not a person’s native language. The term refers to any language (also a third or fourth language) learned after the native language(s) has been acquired. A second language is usually learned as a foreign language. Compared to native language acquisition, nonnative language learning is remarkably associated with emotions. Anxiety has been the most commonly studied emotion in the context of nonnative language learning (Dewaele and MacIntyre, 2014). The term *foreign language anxiety* (FLA) can be defined as “the worry and negative emotional reaction aroused when learning or using a second language” (MacIntyre, 1999, p. 27). Language teachers often observe learners struggling with physical signs of anxiety such as tense muscles, trembling, and dry throat (Oxford, 2017b). Researchers have found that FLA interferes with thoughts, communication, and learning (see a summary by MacIntyre, 2017) using situation-specific scales such as the Foreign Language Classroom Anxiety Scale (FLCAS; Horwitz et al., 1986). Moreover, FLA can wreck the best teaching techniques and render the most attractive material inadequate (Arnold and Brown, 1999). Several suggestions, such as creating a positive, friendly, and relaxed attitude toward students, have been made to improve L2 teaching (Young, 1990). Many learning techniques have also been suggested; these include relaxing, deep breathing, meditation, listening to soothing music, and making positive statements (Oxford, 1990), and activation of supportive emotions, beliefs, and attitudes toward L2 learning and use (Oxford, 2011). More recently, experimental studies to examine the effectiveness of these previously suggested methods have been conducted. The experimental data in such studies are rather controversial, and there is no general agreement about what aspects of L2 learning can be changed to control FLA. Therefore, in this article, we systematically reviewed experimental studies on FLA.

### Recognition of FLA and Suggestions to Control It Before 2000

Researchers in the field of L2 education began to investigate linguistic and nonlinguistic correlates of FLA using the FLCAS. The literature has documented the relationship of FLA to low achievement in L2 listening, speaking, reading, and writing outcome scores (see a literature review by Horwitz, 2001 and meta-analyses by Zhang, 2019 and Botes et al., 2020), to detrimental cognition such as increased self-related beliefs (see an overview by MacIntyre, 2017), and to unfavorable social attitudes and behaviors, such as reduced linguistic self-confidence (see a summary by MacIntyre, 2017). Data from past studies suggest that effects of FLA can be “quite insidious” (Dewaele and MacIntyre, 2014, p. 238); therefore, the importance of coping with FLA is widely recognized. In fact, the pioneering study of Horwitz et al. (1986) on FLA, which led to development of the well-known FLCAS, involved students who specifically sought assistance in reducing L2 classroom anxiety from teachers and counselors. The authors pointed out the importance of L2 learners’ recognizing, coping with, and overcoming this anxiety.

Before the 2000s, most studies made suggestions about classroom interventions that teachers could implement. For example, Horwitz et al. (1986) suggested specific techniques for teachers such as behavioral contracts, relaxation exercises, advice on effective language learning strategies, and journal keeping. Foss and Reitzel (1988) introduced rational emotive therapy to the field of L2 education. This brief psychotherapy is based on the assumption that irrational beliefs are the source of anxiety. Rational emotive therapy helps learners recognize their own self-defeating, irrational beliefs and modify them to more realistic expectations to manage FLA. Koch and Terrell (1991) found pair/group work made their students feel more comfortable than did other activities and thus suggested separating the class into pairs or small groups to help students reduce FLA. Young (1990) recommended that L2 teachers create and maintain a positive, friendly, and relaxed attitude toward students based on her questionnaire results. Oxford (1990) proposed a set of learning tactics called affective strategies to help learners cope with emotional difficulties. Oxford’s affective strategies include progressive (muscle) relaxation, meditation, use of music, making positive statements to oneself (often referred to as positive self-talk), and discussing one’s feelings with someone.

### Empirical Studies on Controlling FLA After 2000

Since the 2000s, a variety of empirical studies on foreign language anxiety reduction (FLAR) have examined different methods, approaches, and contexts. For example, the study of Kondo and Yang (2004) with 202 Japanese undergraduates identified 70 tactics to cope with FLA and categorized them into five strategies. With a different sample of 60 students, Kondo and Yang (2004) further investigated the application of the five strategies, with preparation being used the most at 60.4%, followed by resignation (28.2%), positive thinking (26.2%), relaxation (11.9%), and peer seeking (11.4%). However, they revealed insignificant correlations between FLA scores and frequency of use of the coping strategies. On the other hand, Kao and Craigie (2013) reported that greater use of a positive thinking strategy was related to a lower level of L2 classroom anxiety. Examining 145 distance learning students in the United Kingdom, Hauck and Hurd (2005) reported that 48 (37%) used some strategies to deal with FLA. Among 11 strategies, the three used most frequently were active self-encouragement to take risks (87.5%), use of positive self-talk (64.6%), and imagining a friendly informal chat when speaking in front of others (35.4%) (Hauck and Hurd, 2005). Moreover, Tsiplakides and Keramida (2009) conducted a qualitative case study with 15 students at a secondary school in Greece. They suggested that incorporating short-term project work could be effective for FLAR since it offers a nonthreatening learning environment. They also pointed out the importance of a supportive classroom atmosphere.

### Classroom Interventions for Controlling FLA

In recent years, there has been increasing focus on the relationship between classroom interventions and FLA. Two types of approaches for this line of studies have been observed. One is computer-mediated communication (CMC) as a nontraditional approach. CMC has provided entirely different modalities of classroom interactions. It allows learners to communicate regardless of time or location. For instance, virtual reality, video chat, and voice chat, as employed in York et al.’s (2020) study, could reduce FLA. Another approach is the application of positive psychology (Dewaele et al., 2019; MacIntyre et al., 2019), which offers various interventions to boost positive emotions while alleviating anxiety (Oxford, 2017a). For instance, positive self-talk, as employed in Toyama and Yamazaki’s (2019) study, can help anxious L2 learners “feel more confident in learning the new language” (Oxford, 1990, p. 143). Gregersen (2013) explained that positive psychology could be beneficial for L2 teachers and learners, who can capitalize on positive affect while mitigating the effect of negative emotions such as anxiety. Also, it is important to integrate linguistic and nonlinguistic aspects into L2 education (MacIntyre et al., 2019). Applying positive psychology interventions in class, L2 teachers should deal with learners’ psychological and social aspects (i.e., well-beingness) in addition to linguistic knowledge and skills.

Experimental studies have developed and implemented classroom interventions and evaluated their impact on FLA reduction. However, the classroom interventions’ various characteristics make it difficult to synthesize the findings and apply them to practice. Unfortunately, no previous study has systematically reviewed the various classroom experimental interventions related to FLAR. Teachers may wonder if there is any evidence that a particular FLAR method is effective, how it works, or what can be improved. They may want to know which intervention is the most appropriate for application in their classes. This lack of understanding poses a significant challenge to L2 research and education. Thus, we aimed to systematically review past experimental studies, focusing on various educational interventions and their influence on FLAR.

### Study Approach

This study presents a systematic review of FLAR intervention studies with a narrative approach. A systematic review is a particular type of literature review (Siddaway et al., 2019) that involves “a clearly formulated question” and “uses systematic and explicit methods to identify, select, and critically appraise relevant research, and to collect and analyze data from the studies that are included in the review” (Cochrane Collaboration, 2003, as cited in Siddaway et al., 2019, p. 751). A narrative approach relies “primarily on the use of words and text to summarize and explain the findings”; this approach is considered helpful in “the initial stages of a review” (Popay et al., 2006, p. 5). Moreover, it can be used when the experimental and quasi-experimental studies included in the review are not sufficiently similar to allow a meta-analysis (Mays et al., 2005). We adopted a narrative approach to a systematic review in this study since we aimed to systematically and transparently collect quantitative studies that have used diverse methodologies and to offer a text-based synthesis and analysis rather than a statistical summary.

Since a systematic review allows us to replicate studies, our methodology using a systematic review is congruent with a direction based on the view of King and Mackey (2016). They illustrated the importance of replication studies in the field of applied linguistics. In the field of L2 learning and teaching, a systematic review with a descriptive or narrative presentation of findings can be seen in the study of Boo et al. (2015), who utilized databases and anthology chapters from 2005 to 2014 on L2 motivation. We also reviewed processes and strategies from several other studies that applied a systematic review with meta-analysis in the field—covering the relationships between FLA and achievement (Teimouri et al., 2019), between FLA and L2 performance (Zhang, 2019), and between FLCA and academic achievement (Botes et al., 2020).

In this study, the term *foreign language anxiety* is used to refer to “the worry and negative emotional reaction aroused when learning or using a second language” (MacIntyre, 1999, p. 27). The term *educational intervention* and *classroom intervention* are used to refer to any activity, strategy, or method used to teach a new skill. The terms *experimental study* and *intervention study* are used to refer to studies where researchers introduced an intervention, collected data, and observed the effects of the intervention. The terms *FLAR interventions* and *FLAR methods* are used to refer to any educational interventions related to FLA management.

The following sections present the methodology for the systematic review, highlight the trends and patterns that were revealed, and discuss the findings in connection with previous research.

## Methodology

### Study Guideline

To develop a process for a systematic review with a narrative approach, we relied mainly on four studies on systematic reviews from Siddaway et al. (2019); Jiménez et al. (2020), Pluye and Hong (2014), and Resurrección et al. (2014). The first study allowed us to develop a theoretical framework and process for a systematic review with a narrative approach, while the other three studies provided useful insight. Also, we followed the overall flow of PRISMA (Moher et al., 2009).

### Pilot Search

Prior to the literature search, we performed a pilot search to gain initial insight on the output of the database system, gage our study’s relevance, and help us develop appropriate inclusion and exclusion criteria when entering several search term candidates. The first and second authors of this study independently carried out a pilot search. For this pilot search, we used the keywords of “foreign language anxiety” AND “reduction” in a search of ProQuest. The database allowed us to select publications written in the English language, with no time boundary, and among the document/publication types of article, book chapter, and book. To determine publication type, we referred to the review study of Boo et al. (2015), which considered only publications from journals through a database search and reviewed book chapters of seven specific anthologies published in the past. This pilot search resulted in 51 studies that were considered to be a pool for potential literature for our study review purpose.

### Literature Search

To find potential studies relevant to FLAR, we utilized five online databases: Scopus, Linguistics and Language Behavior Abstracts, PsycINFO, PsycArticles, and PTSDpubs. All databases except for Scopus were accessed via ProQuest, a platform for searching multiple databases across multiple disciplines. Searches were performed using the key term “foreign language anxiety” combined with (“AND”) each of nine different secondary terms: “reduction,” “reducing,” “decrease,” “decreasing,” “lowering,” “relieving,” “relief,” “alleviate,” and “alleviating.” Different forms of the same word (e.g., *reduction* and *reducing*) were used in an attempt to capture more articles. The search using the databases was conducted in mid February 2020, without time limitations among the publications of article, book chapter, and book in the English language, and was last updated on November 28, 2020. The first and second authors searched the databases independently and then confirmed whether their database search results were consistent.

### Inclusion and Exclusion Criteria

To identify studies relevant to our research questions, we established six inclusion and exclusion criteria (Table 1). Included studies had to be intervention/experimental studies that used a scale relevant to FLA and presented quantitative results; systematic reviews and meta-analysis studies were excluded. The studies also had to be published in English as a journal article, book chapter, or book. Studies from the search results that did not meet these conditions were excluded from our review.

**TABLE 1:** Inclusion and exclusion criteria for review.

Category	Inclusion criteria	Exclusion criteria
1. Study type	Intervention/experimental study	Not intervention/experimental study
2. Study design	Not systematic review or meta-analysis	Systematic review or meta-analysis
3. Scale description	Description/explanation of scale relevant to FLA	No description/explanation of scale
4. Result description	Quantitative results	Only qualitative results
5. Language	English	Not English
6. Publication type	Article, book chapter, or book	Conference proceedings, university-specific publication, working paper, or dissertation/thesis

*FLA, foreign language anxiety.*

### Data Extraction

**FIGURE 1:**
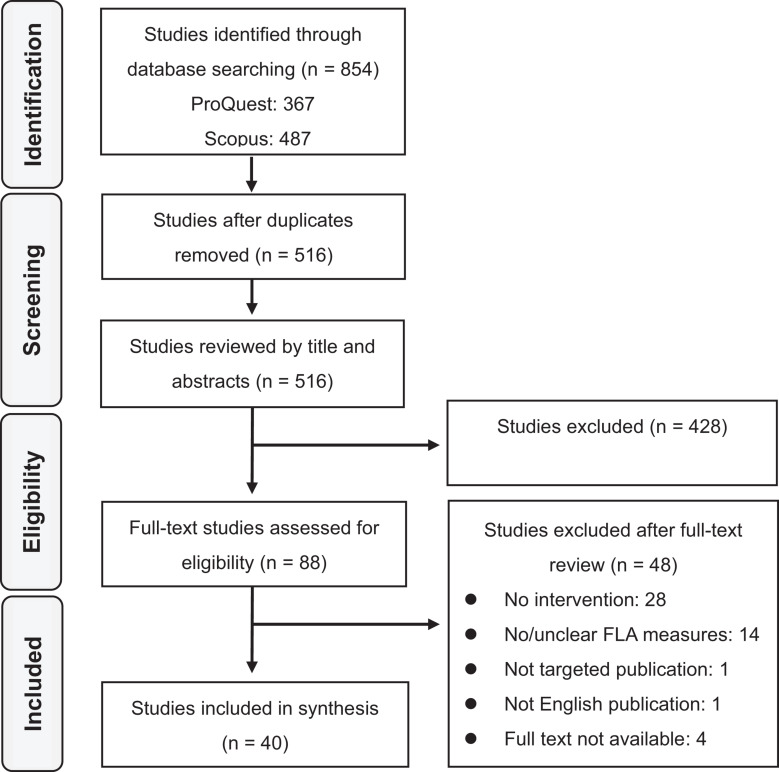
PRISMA flowchart of the FLAR studies included.

The first database search identified 854 potentially eligible studies: 367 from ProQuest and 487 from Scopus. After removal of 338 duplicates, 516 potential studies were reviewed by title and abstract. The first and second authors analyzed the titles and abstracts separately. Then, they discussed results of reviewed studies and excluded 428 studies, leading to 88 studies to assess for eligibility based on full-text analysis. These two authors read the 88 full-text studies and independently analyzed whether they met the inclusion/exclusion criteria. After independent analysis, the authors discussed results to determine studies to be included in the synthesis. The full-text analysis led to the elimination of 48 studies: 28 because of lack of intervention/experimental study, 14 because of lack of FLA measure or unclear measures, one because it was not in a targeted publication, one because it was not an English publication, and four because of lack of availability of full text. Accordingly, 40 studies were included in the synthesis based on the criteria. Figure 1 reviews the flow of data extraction using the PRISMA diagram (Moher et al., 2009).

Various characteristics of the included studies were documented, including publication information (author and year of publication), study design characteristics (sample, intervention type, duration, and scale), and statistical characteristics (analytical methods and key results). Table 2 lists the included studies with their characteristics and FLAR interventions.

**TABLE 2:** Descriptive classification of FLAR intervention characteristics.

Publication information	Study design characteristics				Statistical characteristics	
References	Sample	Intervention	Duration	Scale	Analytical method	Key results
Abood and Abu-Melhim, 2015	EG (*n* = 16), CG (*n* = 16), UG, Jordan	RET (peer and teacher feedback, positive self-talk)	5 weeks	FLCAS	Two-way ANOVA	Sig between-group difference at posttest but not at pretest
Alrabai, 2015	EG (*n* = 236), CG (*n* = 232), HS, UG, Saudi Arabia	Teacher’s FLAR strategies	8 weeks	FLCAS (α = 0.93)	Repeated measures ANOVA, ANCOVA	Sig reduction only in EG, n.s. between-group difference at pretest, main effect of intervention
Arnold, 2007	EG1 (*n* = 21), EG2 (*n* = 23), CG (*n* = 12), UG, USA	CMC (text chat and online forums)	1 semester	FLCAS^1^	Repeated measures ANOVA	Sig reduction in all groups, n.s. between-group difference
Bailey and Cassidy, 2019	EG (*n* = 41), No CG, UG, South Korea	Peer feedback	15 weeks	SLWAI (α ≥ 0.77)	Paired *t*-test	n.s. reduction
Baralt and Gurzynski-Weiss, 2011	EG (*n* = 25), No CG, UG, USA	CMC (text chat)	1 session	FLSAQ (α ≥ 0.83)	Paired *t*-test, repeated measures ANOVA	n.s. reduction
Bashori et al., 2020	EG (*n* = 167), No CG, HS, Indonesia	Web-based individual L2 skill training	3 weeks	FLCAS^1^ (α = 0.80)	Paired *t*-test	n.s. reduction
Bielak, 2018	EG (*n* = 15), CG (*n* = 8), UG, Poland	Strategy instruction	9 months	FLCAS (α ≥ 0.86)	Wilcoxon SRT, MWU test	Sig reduction only in EG, sig between-group difference at pretest but not posttest
Çapan, 2013	EG (*n* = 18), No CG, UG, Turkey	CMC (video chat)	8 virtual meetings	FLCAS	Wilcoxon SRT	Sig reduction
Chen and Lee, 2011	EG (*n* = 4), No CG, HS, Taiwan	1:1 teacher feedback using an emotion-recognition system	1 session	FLCAS^1^	Descriptive analysis	Reduced, but no statistical test
Chen et al., 2016	EG1 (*n* = 27), EG2 (*n* = 30), CG (*n* = 31), UG, Taiwan	Collaborative reading and teacher feedback	50 min	FLRAS	ANCOVA	Sig difference between CG and EG2 (collaborative reading and teacher feedback)
Foroutan and Noordin, 2012	EG (*n* = 22), CG (*n* = 20), UG, Malaysia	CMC (dialogue journal writing using e-mail)	7 weeks	SLWAI (α = 0.86)	Paired *t*-test, Ind *t*-test	n.s. reduction in both groups, n.s. between-group difference at pretest and posttest
Galante, 2018	EG (*n* = 13), CG (*n* = 11), PI, Brazil	Drama	4 months	FLCAS^1^ (α = 0.77, 0.88)	Repeated measures ANOVA	Sig reduction in all groups, n.s. between-group difference
Hamzaoğlu and Koçoğlu, 2016	EG (*n* = 15), CG (*n* = 15), HS, Turkey	CMC (podcasting)	12 weeks	FLCAQ (α = 0.81)	Ind *t*-test	Sig between-group difference at posttest
He, 2017	EG (*n* = 30), CG (*n* = 30), UG, China	Strategy instruction	4 months	FLSAQ (α = 0.86)	MANOVA	n.a.
Jahin, 2012	EG (*n* = 20), CG (*n* = 20), UG, Saudi Arabia	Peer reviewing in writing	14 weeks	SLWAI (α = 0.89)	n.a.	Sig reduction only in EG, sig between-group difference at posttest
Jin et al., 2020	EG (*n* = 20), CG (*n* = 22), UG, China	Student behavioral contracts	1 week	ECAS (α ≥ 0.83)	Two-way ANOVA	Sig main effect of time, n.s. main effect of group, sig interaction effect of group and time
Kralova et al., 2018	EG (*n* = 30), CG (*n* = 33), UG, Slovakia	Psychosocial training	24 weeks	FLPAS	Wilcoxon SRT/RST	Sig reduction in both groups, sig between-group difference at posttest but not at pretest
Kralova et al., 2017	EG (*n* = 22), CG (*n* = 46), UG, Slovakia	Psychosocial training	12 weeks	FLPAQ	Wilcoxon SRT/RST	Sig reduction in both groups, sig between-group difference at posttest but not at pretest
Kruk, 2016	EG (*n* = 13), CG (*n* = 14), HS, Poland	CMC (virtual world)	1 week	FLCAS (α ≥ 0.89)	Wilcoxon SRT, MWU test	n.s. between-group difference, n.s. reduction in both groups
Ku and Chen, 2015	EG (*n* = 49), No CG, HS, Taiwan	CMC (collaborative learning with Google Wiki)	3 days	TCLAI (α = 0.91)	Paired *t*-test	Sig reduction
Kwiecień-Niedziela et al., 2020	EG (*n* = 17), CG (*n* = 25), MS, Poland	Drama	n.a.	FLCAS	Ind *t*-test	Sig between-group difference at posttest
Liao and Wang, 2015	EG (*n* = 40), CG (*n* = 34), UG, Taiwan	Feminist pedagogical learning	12 weeks	ECAS (α ≥ 0.86)	Ind *t*-test	Sig between-group difference at posttest but not at pretest
Liu et al., 2018	EG (*n* = 27), CG (*n* = 28), PS, Taiwan	Performance (cooperative digital storytelling)	10 weeks	FLCAS	ANCOVA	Sig reduction only in EG, sig between-group difference at posttest
Melchor-Couto, 2017	EG (*n* = 7), CG (*n* = 7), UG, United Kingdom	CMC (virtual world)	4 sessions	FLCAS^1^	ANOVA	Sig difference in EG between sessions, but n.s. in CG
Mostafavi and Vahdany, 2016	EG (*n* = 30), CG (*n* = 30), HS, Iran	Strategy instruction	6 weeks	FLCAS^1^ (α = 0.87)	Paired *t*-test, Ind *t*-test	n.s. reduction in both groups, n.s. between-group difference at pretest, but EG had higher scores than CG at posttest
Nosratinia and Abdi, 2017	EG (*n* = 35), CG (*n* = 35), UG, Iran	Portfolio assessment	5 weeks	FLCAS (α = 0.70)	Ind *t*-test	Sig between-group difference at posttest
Sağlamel and Kayaoğlu, 2013	EG (*n* = 22), No CG, UG, Turkey	Creative drama	6 weeks	FLCAS^1^ (α = 0.90)	Wilcoxon SRT	Sig reduction
Scida and Jones, 2017	EG (*n* = 95+79), CG (*n* = 115+82), UG, United States	Contemplative practices	1 semester	FLCAS	Paired *t*-test, Ind *t*-test	Sig reduction in both groups, n.s. between-group difference at posttest
Shimbo, 2008	EG (*n* = 25), CG (*n* = 29), UG, Australia	Music, relaxation, and suggestions	12 weeks	FLCAS	Paired *t*-test	n.s. reduction
Soleimanirad and Shangarffam, 2016	EG (*n* = 30), CG (*n* = 30), LI, Iran	Collaborative reasoning	18 sessions	FLCAS	ANCOVA	Sig between-group difference at posttest
Tang, 2016	EG (*n* = 58), CG (*n* = 57), UG, China	Formative assessment	9 months	ECSAS	Paired *t*-test, Ind *t*-test	Sig reduction in EG, sig between-group difference at posttest but not at pretest
Toyama and Yamazaki, 2019	EG (*n* = 57), CG (*n* = 70), UG, Japan	RET (peer and teacher feedback, positive self-talk)	6 weeks	FLCAS (α = 0.93)	Paired *t*-test, Ind *t*-test	Sig reduction only in EG, sig between-group difference at pretest but not posttest
Tsiriotakis et al., 2017	EG (*n* = 4 groups), CG (*n* = 4 groups): each group had 20–25, PS, Greece	Strategy instruction	17 weeks	SLWAI (α ≥ 0.77)	Repeated measures MANOVA	Sig reduction only in EG, n.s. between-group difference at present, main effect of intervention
Uştuk and Aydın, 2018	EG (*n* = 40), No CG, UG, Turkey	Paralinguistic feature instruction and drama	4 weeks	FLCAS (α ≥ 0.82)	Paired *t*-test	Sig reduction partly
Wei et al., 2018	EG1 (*n* = 30), EG2 (*n* = 30), EG3 (*n* = 30), CG (*n* = 30), UG, Taiwan	Competitive gaming and personalized assistance	30 min	FLRAS^1^ (α = 0.86)	Two-way ANOVA	Sig difference between groups with competitive gaming and the others
Xiangming et al., 2020	EG (*n* = 158), No CG, PG, China	CMC (Rain Classroom)	10 weeks	ELCAS	Paired *t*-test	Sig reduction
Yayli, 2017	EG (*n* = 93), No CG, UG, Turkey	Group work	1 semester	FLCAS, FLLAS (α = 0.91, 0.90)	Paired *t*-test	n.s. reduction about both measures
York et al., 2020	EG (*n* = 30) but n.a. of each three-groups, UG & GS, Japan	CMC (virtual reality)	1 session	FLCAS^1^	Repeated measures ANOVA, paired *t*-test	Sig reduction in all groups, n.s. between-group difference
Yu et al., 2020	EG (*n* = 17), No CG, UG, Taiwan	CMC (virtual world)	9 weeks (5 sessions)	FLCAS (α ≥ 0.86)	Wilcoxon SRT	n.s. reduction
Zarrinabadi and Rezazadeh, 2020	EG (*n* = 210), No CG, PI, Iran	Feedback, feed up, feed forward	12 sessions	SLWAI (α ≥ 0.79)	MANOVA	Sig reduction of combination of feedback types

*1:1, one-to-one; ANCOVA, analysis of covariance; ANOVA, analysis of variance; CG, control group; CMC, computer-mediated communication; ECAS, English Classroom Anxiety Scale; ECSAS, English Classroom Speaking Anxiety Scale; ELCAS, English Language Classroom Anxiety Scale; EG, experimental group; FLAR, foreign language anxiety reduction; FLCAQ, Foreign Language Classroom Anxiety Questionnaire; FLCAS, Foreign Language Classroom Anxiety Scale; FLCAS^1^, a modified version of FLCAS; FLLAS, Foreign Language Listening Anxiety Scale; FLPAQ, Foreign Language Pronunciation Anxiety Questionnaire; FLPAS, Foreign Language Pronunciation Scale; FLRAS^1^, a modified version of Foreign Language Reading Anxiety Scale; FLSAQ, Foreign Language State Anxiety Questionnaire; GS, graduate school; HS, high school; Ind, independent; LI, language institute; LMS, learning management system; MANOVA, multivariate analysis of variance; MS, middle school; MWU, Mann–Whitney U; n.a., not applicable or insufficient description; n.s., not significant; PI, private institute; PG, postgraduates; PS, primary school students; RET, rational emotive therapy; RST, rank sum test; sig., significant; SLWAI, Second Language Writing Anxiety Inventory; SRT, signed rank test; TCLAI, Transnational Collaborative Learning Anxiety; UG, undergraduates; α, Cronbach’s alpha.*

## Results

### Synthesis of Results

Our database search and extraction identified 40 studies that assessed FLAR intervention. Before analyzing key features of effective FLAR intervention, we conducted descriptive analysis to summarize trends of FLAR interventions in the literature.

As depicted in Table 3, the earliest experimental FLAR research identified in this study was published in 2007, and the latest in November 2020. The number of publications on FLAR interventions increased over that time period, except for 2019. Additionally, since FLAR was already being addressed in the late 1980s and early 1990s (see Foss and Reitzel, 1988; Young, 1992), the total number of 40 publications was smaller than might have been expected. Studies came out of 17 countries/regions, but half were performed in four countries/regions: Taiwan (*n* = 7), Turkey (*n* = 5), China (*n* = 4), and Iran (*n* = 4); further, over two-thirds (*n* = 28) were conducted in East and West Asia.

**TABLE 3:** Characteristics of the 40 studies included in the systematic review.

Category	Variable	*n*	Variable	*n*
Publication year	2007	1	2016	6
	2008	1	2017	7
	2011	2	2018	6
	2012	2	2019	2
	2013	2	2020	7
	2015	4		
Country/region	Taiwan	7	Australia	1
	Turkey	5	Brazil	1
	China	4	Greece	1
	Iran	4	Indonesia	1
	Poland	3	Jordan	1
	United States	3	Malaysia	1
	Japan	2	South Korea	1
	Saudi Arabia	2	United Kingdom	1
	Slovakia	2		
Educational institution	Primary school	2	Graduate school	1
	Middle school	1	Postgraduates	1
	High school	7	Private institute	2
	Undergraduate	27	Language institute	1
Sample size	9 or fewer	1	50–99	12
	10–49	18	100 or over	9
Control group	Yes	27	No	13
Duration^a^	1 h or less	2	10–19 weeks	14
	3 days	1	20–36 weeks	3
	1–9 weeks	12	Other	8
Scale^b^	FLCAS	23	ELCAS	1
	SLWAI	5	FLCAQ	1
	FLSAQ	2	FLLAS	1
	FLRAS	2	FLPAQ	1
	ECAS	2	FLPAS	1
	ECSAS	1	TCLAI	1
Features of interventions^c^	CMC	13	Strategy	5
	Student–student interaction	12	Counseling and training	4
	Teacher-student interaction	11	Mood boosters	4
	Performance	5		
Analytical methods^d^	Paired *t*-test	14	Wilcoxon RST	2
	Independent *t*-test	9	MANOVA	2
	Wilcoxon SRT	7	ANOVA	1
	Repeated ANOVA	5	Repeated MANOVA	1
	ANCOVA	4	Descriptive statistics only	1
	Two-way ANOVA	3	not reported	1
	Mann–Whitney U test	2		

*^a^One semester was converted to 17 weeks. Nine months was converted to 36 weeks.^b^Cronbach’s alphas reported: 11 for FLCAS, 5 for SLWAI, 2 for ECAS, 2 for FLSAQ, and 1 each for FLCAQ, FLLAS, FLRAS, and TCLAI.^c^Some studies used more than one intervention.^d^Some studies used more than one method.CMC, computer-mediated communication; ECAS, English Classroom Anxiety Scale; ECSAS, English Classroom Speaking Anxiety Scale; ELCAS, English Language Classroom Anxiety Scale; FLCAQ, Foreign Language Classroom Anxiety Questionnaire; FLCAS, Foreign Language Classroom Anxiety Scale; FLLAS, Foreign Language Listening Anxiety Scale; FLPAQ, Foreign Language Pronunciation Anxiety Questionnaire; FLPAS, Foreign Language Pronunciation Scale; FLRAS, Foreign Language Reading Anxiety Scale; FLSAQ, Foreign Language State Anxiety Questionnaire; SLWAI, Second Language Writing Anxiety Inventory; TCLAI, Transnational Collaborative Learning Anxiety.*

Foreign language anxiety reduction interventions were more common in higher education institutions than in primary schools. As shown in Table 3, most studies took place in undergraduate schools (*n* = 27) followed by high schools (*n* = 7). Three-fourths of the FLAR intervention studies had sample sizes smaller than 100; 18 had 10–49 students, and 12 had 50–99 students. One study, that of Alrabai (2015), involved 468 students. More than two-thirds of the studies had a control group (*n* = 27), while one-third had only an experimental group (*n* = 13). The shortest duration of the interventions was 1 h or less, while the longest was 9 months, equivalent to 36 weeks. Almost one-third took place between 1 and 9 weeks (*n* = 12) or between 10 and 19 weeks (*n* = 14). Twelve different scales were used to measure the effectiveness of interventions. Among the 40 studies, the FLCAS (Horwitz et al., 1986) was most widely used (*n* = 23). Another popular scale was the Second Language Writing Anxiety Inventory (SLWAI) (Cheng, 2004) (*n* = 5). One explanation for the popularity of these scales is that L2 speaking and writing are more likely to cause FLA than reading and listening are. Other scales similar to the FLCAS were developed or modified among this group of studies, including the English Classroom Anxiety Scale (ECAS) (Liao and Wang, 2015), English Classroom Speaking Anxiety Scale (ECSAS) (Tang, 2016), English Language Classroom Anxiety Scale (ELCAS) (Liu, 2009), and Foreign Language Classroom Anxiety Questionnaire (FLCAQ) (Young, 1990; Woodrow, 2006). The use of those scales also indicated a focus on L2 speaking anxiety. Sixty percent of the studies reported Cronbach’s alphas (*n* = 24).

With regard to statistical methods of the FLAR intervention studies, almost all studies conducted statistical tests, while one study performed descriptive analysis (Chen and Lee, 2011) and one did not clearly present a statistical method (Jahin, 2012). Over one-third of studies used a paired *t*-test (*n* = 14) followed by an independent *t*-test (*n* = 9), Wilcoxon signed rank test (*n* = 7), and repeated analysis of variance (*n* = 5).

Several interventions included more than one task/tactic/tool for L2 education; thus, we use the term *feature* to differentiate the types of reviewed interventions. We found seven features: CMC, student–student communication, teacher-student communication, performance, strategy instruction, counseling and training, and mood boosters. The most common features were related to communication: CMC such as *text chat* and *online forums* (Arnold, 2007), student–student interactions such as *peer feedback* (Bailey and Cassidy, 2019), and teacher-student interaction such as *portfolio assessment* (Nosratinia and Abdi, 2017). Some interventions included both student–student and teacher-student interactions (Liao and Wang, 2015; Chen et al., 2016; Tang, 2016), and some had both CMC and student–student/teacher-student communication (e.g., Ku and Chen, 2015). Our review further found learner-internal features of FLAR interventions. Five studies used performance such as drama (Sağlamel and Kayaoğlu, 2013; Galante, 2018; Uştuk and Aydın, 2018; Kwiecień-Niedziela et al., 2020) or digital storytelling (Liu et al., 2018), five involved strategies (Alrabai, 2015; Mostafavi and Vahdany, 2016; He, 2017; Tsiriotakis et al., 2017; Bielak, 2018), four interventions used counseling and training (Abood and Abu-Melhim, 2015; Kralova et al., 2017, 2018; Toyama and Yamazaki, 2019), and four used mood boosters such as meditation (Scida and Jones, 2017), music (Shimbo, 2008), gaming (Wei et al., 2018), and web-based L2 exercises (Bashori et al., 2020).

### Types of Interventions

**FIGURE 2:**
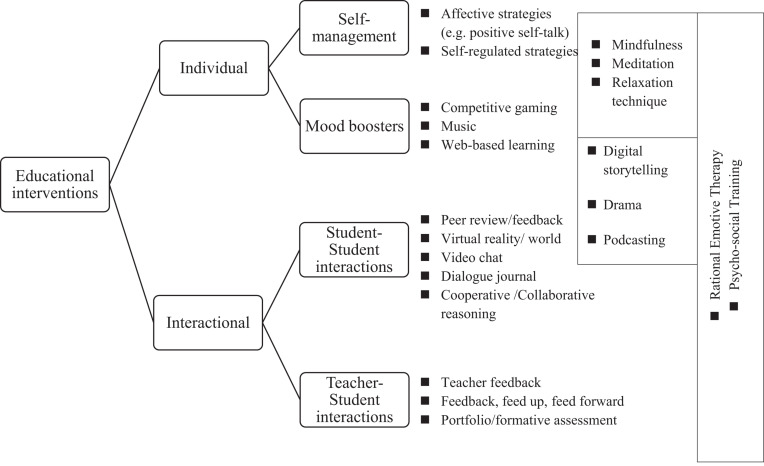
Types of foreign language anxiety reduction (FLAR) methods.

The educational interventions reviewed in this article have the potential to affect individual (learner-internal) and interactional dimensions in L2 learning. Figure 2 shows the types of educational interventions. The types on the left are based on our review results. Examples of interventions are listed on the right. Those that can affect more than one dimension are placed between the two dimensions. Each major type (individual and interactional) is divided into two more subtypes: self-management, mood-boosters, student–student interactions, and teacher-student interactions (Figure 2). The following sections summarize the main results obtained with each type and subtype.

#### The Individual Type

As shown in Figure 2, the reviewed interventions that can change learners’ individual dimensions are categorized into two groups: *self-management* and *mood boosters*. This distinction is useful because some methods (e.g., affective strategy instruction) help learners develop their self-management capabilities, while others assist mood improvement (e.g., music and gaming). The following paragraphs first compare and contrast various methods developing L2 learners’ self-management skills and then analyze those improving learners’ mood.

Our review found two interventions that have an effect on individuals’ self-management capabilities. Use of strategies was frequently reported, but strategy interventions were highly heterogeneous. Some studies used Oxford (1990, 2011, 2017a) *affective strategies* that target L2 learners who experience negative effects of anxiety. By teaching affective strategies, language teachers can help learners develop their emotion management capabilities (Oxford, 2015). Bielak (2018) confirmed the effectiveness of affective strategy instruction with significantly lower level of FLCA and more frequent and wider range of affective strategy use in the intervention condition. Mostafavi and Vahdany (2016) also reported the effectiveness of affective strategy instruction; however, the FLCAS scores of their intervention group somehow increased. Positive self-talk or “self-encouragement via positive statements” (Oxford, 1990, p. 142), as employed in Toyama and Yamazaki’s (2019) study, is often considered a type of affective strategy. Toyama and Yamazaki (2019) used positive self-talk in the framework of rational emotive therapy and reported within- and between-group decreases in FLA. Tsiriotakis et al. (2017) implemented Graham and Harris’s (1989) *self-regulated strategy* development, which aims to enhance affective, behavioral, and cognitive aspects of learning, and confirmed its effectiveness in reducing L2 writing anxiety as well as improving L2 skills. The others used a range of questionnaire survey-based strategies (He, 2017) or an assorted set of strategies taken from previous literature (Alrabai, 2015). A criticism of these studies is that they used a range of strategies for teachers and learners, and thus it is difficult to determine which strategy or which set of strategies was effective in FLA reduction.

As shown in Figure 2, contemplative practices and relaxation were placed between self-management and mood boosters since these methods work on thoughts and feelings. Results concerning contemplative practices (Scida and Jones, 2017) and relaxation (Shimbo, 2008) remain unclear. Psychologists have demonstrated the effects of contemplative practices such as mindfulness and meditation on physical and psychological health and well-being (see a review by Dorjee, 2016). Similarly, relaxation techniques reduce frustration and boost confidence (MacIntyre and Gregersen, 2012). However, these methods require future research in L2 education, as their effectiveness has not yet been confirmed statistically.

Our review also found several interventions that have an effect on the individual’s mood. The use of music, competitive gaming, and web-based learning can be categorized as mood boosters, since these interventions have the potential to bring enjoyment, concentration, or excitement to L2 learners. However, the results were contradictory. While competitive gaming was reported to be effective in reducing FLA (Wei et al., 2018), the effectiveness of music (Shimbo, 2008) and web-based learning (Bashori et al., 2020) remain unclear.

#### The Interactional Type

Interactional interventions can be categorized into two groups: student–student and teacher-student interactions (Figure 2). Different studies employed an intervention to enhance both types of interactions and confirmed a significant between-group difference at posttest (Liao and Wang, 2015; Chen et al., 2016; Soleimanirad and Shangarffam, 2016; Tang, 2016). Jin et al. (2020) used a behavioral contract method where L2 learners signed a contract to commit to speaking in L2 class and confirmed a significant decrease in the intervention condition compared to the control group. Enhancing only student–student communication may not result in FLA reduction. For instance, peer feedback (Bailey and Cassidy, 2019) and group work (Yayli, 2017) were not effective for FLAR. The effectiveness of peer reviewing in L2 writing (Jahin, 2012) remained unclear because of insufficient statistical reports. In contrast, a focus on teacher-student interaction enhancement is likely to be effective. Portfolio assessment, which enhances teacher-student communication, was found effective in reducing more FLA in comparison with a non-interventional group (Nosratinia and Abdi, 2017). Teacher feedback, feed up, and feed forward (Zarrinabadi and Rezazadeh, 2020) also resulted in a significant reduction in FLA, but the study had no comparison group. Promoting teacher feedback using an emotion-recognition system decreased FLA (Chen and Lee, 2011); however, the authors did not report statistical results.

Results of CMC remain unclear. Only two studies compared intervention and nonintervention groups and found a significant difference. Hamzaoğlu and Koçoğlu (2016) used a podcasting intervention and showed that the intervention group achieved not only lower anxiety but also higher speaking test scores. Use of the virtual world called *Second Life* for weekly conversation training for 4 weeks also resulted in a significant FLA decrease only in the intervention condition (Melchor-Couto, 2017). In the virtual world, interactions are achieved through avatars. Two other studies with the virtual world, however, did not find any significant differences: *Second Life* in Yu et al. (2020) and *Yoowalk* for grammar practice in speaking for two classes within a week (Kruk, 2016). York et al. (2020) used a virtual reality where users could interact using their avatars, voice and paralinguistic cues in gestures. In their experiment, participants completed a spot-the-difference task in three different synchronous CMC modes: voice chat, video chat, and virtual reality with voice chat. All three modes successfully reduced FLA, but no between-group difference was found. We found many studies with various CMC interventions designed to enhance student–student and/or teacher-student interactions. In Arnold’s (2007) experiment, student–student interactions through text chat, online forums, and face-to-face interaction were compared. FLA was reduced in all groups, but no between-group difference was found. Using email for dialogue journal writing to enhance student–student interactions (Foroutan and Noordin, 2012) and synchronous text chat between a teacher and a student (Baralt and Gurzynski-Weiss, 2011) were not effective in reducing FLA. Many other studies did not use a control group. Çapan (2013) enhanced interactions between students in different institutions using video chat and showed a significantly lower FLCAS scores after the intervention, but there was no comparison group. Xiangming et al. (2020) used *Rain Classroom*, a mobile application for teaching and learning, and found not only a significant decrease in ELCAS scores but also significant increases in L2 speaking, listening, and writing test scores after the intervention. Although they did not have a control group, they succeeded in improving both linguistic and nonlinguistic aspects in L2 learning. Use of Google Wiki for cross-cultural collaborative learning was also effective in reducing FLA, but there was no control group (Ku and Chen, 2015).

#### Performance Type

Some methods reviewed in this study improved both individual and interactional dimensions in L2 learning. Interventions including performance such as drama or digital storytelling are grouped into this type. We found four studies with the drama technique (Sağlamel and Kayaoğlu, 2013; Galante, 2018; Uştuk and Aydın, 2018; Kwiecień-Niedziela et al., 2020) and one with cooperative digital storytelling (Liu et al., 2018). The effectiveness of drama intervention remains unclear. None of the drama intervention studies showed a significant within- and between-group difference; we found only partial evidence. In contrast, the study of Liu et al. (2018) is an excellent example of an intervention that can boost personal mood and enhance student–student communication. They hypothesized that students working cooperatively would gain greater knowledge and less FLA than those working individually. Both cooperative and individualistic learning groups engaged in a digital storytelling task in their study. They showed that the cooperative learning group achieved not only lower anxiety but also higher test scores than the individualistic learning group did at the posttest. Surprisingly, those engaged in the digital storytelling task individually increased their FLCA level by the end of the experiment. Podcasting (Hamzaoğlu and Koçoğlu, 2016) described in the previous section concerning CMC also includes a performance phase. In Figure 2, these methods are placed between individual and interactional. The preparation and performance of these activities can be a mood booster. In addition, the preparation or practice phase of drama or cooperative storytelling enhances student–student interactions.

#### Training/Counseling Type

Training/counseling interventions can also improve both individual and interactional dimensions of L2 learning. In fact, they are designed to improve student–student interactions through peer feedback and teacher-student interaction through debriefing and feedback. Applying rational emotive therapy combined with positive self-talk, two studies (Abood and Abu-Melhim, 2015; Toyama and Yamazaki, 2019) reported successful decrease in FLA.

Use of psychosocial training (Kralova et al., 2017, 2018) also resulted in significantly more FLA reduction in the intervention condition. Kralova et al.’s (2017) psychosocial training was conducted in smaller groups with a psychologist and placed importance on both teacher-student and student–student interactions. The psychologist’s goal was to create a supportive environment and an atmosphere that generates psychological trust and helps learners cope with stressful situations, while the learners’ goal was to develop social abilities through sharing ideas on various topics (e.g., who I am, we are all different, communication, emotions, how to resolve conflicts). In their experiment, both intervention and control groups received L2 pronunciation training while only the intervention group received psychosocial training. The results revealed that the intervention group achieved not only higher pronunciation scores but also lower anxiety. Perhaps one criticism of the study is that requiring a psychologist to lead this training reduces the feasibility of its application in L2 education. In Figure 2, these interventions are placed in a box extending perpendicularly, showing their multiple purposes.

## Discussion

The present review has focused on the association between various types of educational interventions and FLA reduction in order to clarify the state of the field and suggest future directions. To the best of our knowledge, this is the first systematic review specifically examining the types and effectiveness of educational interventions on FLA.

Consistent with previous suggestions, affective strategy instruction improved FLA, as measured by the FLCAS (Bielak, 2018). Affective strategies (Oxford, 1990, 2011, 2017a) target L2 learners who experience negative effects of anxiety and help them create positive emotions, beliefs, and attitudes and stay motivated (Oxford, 2011). By teaching affective strategies, language teachers can help learners develop their emotion management capabilities (Oxford, 2015). The cognitive effects of FLA, such as increased self-related cognition (e.g., thoughts of failure and self-deprecating thoughts) (MacIntyre, 2017), can also be managed by means of affective strategies. Strategy instruction can provide anxious L2 learners with “readily sharable techniques and strategies” (Oxford, 2015, p. 385). Therefore, affective strategy instruction can strengthen learners’ ability to cope with FLA, which may affect emotional, cognitive, and attitudinal dimensions in learning. Affective strategy instruction is also in line with Dewaele and MacIntyre (2014, 2016, 2019) suggestion that the main cause of FLA is the learner rather than the teacher. Nevertheless, our systematic review identified only two intervention studies that examined the effectiveness of affective strategy instruction, and only one of them could show a significant FLA reduction in the intervention condition. Moreover, the affective strategies in Oxford (1990) were updated in Oxford (2011) to include eight meta-affective strategies such as Monitoring Affect. While Bielak (2018) taught both affective and meta-affective strategies to students, Mostafavi and Vahdany (2016) used only the affective strategies in Oxford (1990). Therefore, further work is needed to compare the effect of affective strategy instruction and meta-affective strategy instruction, respectively.

We also found interventions that combined two or three methods. Rational emotive therapy (Foss and Reitzel, 1988) combined with positive self-talk or “self-encouragement via positive statements” (Oxford, 1990, p. 142) and peer and teacher feedback improved FLA, as measured by the FLCAS (Abood and Abu-Melhim, 2015; Toyama and Yamazaki, 2019). This brief therapy helps learners recognize their own irrational beliefs as a source of anxiety and modify them to more realistic expectations to manage FLA. In Toyama and Yamazaki’s (2019) intervention, L2 learners were instructed to have positive images of themselves being more confident during positive self-talk. Such instruction or assistance seems necessary for the FLAR process. Their qualitative analysis revealed that participants attempted to “change their negative feelings, perceptions, beliefs, and behaviors” (Toyama and Yamazaki, 2019, p. 9) during positive self-talk activity, and their level of FLA was successfully lowered. This combination can be implemented without much work on instructors (i.e., training or much practice before implementation). Similarly, psychosocial training combined with L2 pronunciation training improved FLA, as measured by the Foreign Language Pronunciation Anxiety Scale (FLPAS) (Kralova et al., 2017). An instructor’s positive, patient, and relaxed attitude (Young, 1990) along with social-skill and pronunciation skill development realized in their psychosocial training should have played a critical role in FLAR. Such multipurpose interventions are placed between the types in Figure 2. Note that this figure is in line with MacIntyre’s (2017) comment: “Anxiety is influenced by internal physiological processes, cognition, and emotional states along with the demands of the situation and the presence of other people, among other things, considered over multiple timescales. Anxiety has both internal and social dimensions” (p. 34).

While reviewing the literature in this field, we identified some interventions combining cooperative learning with digital storytelling (Liu et al., 2018), Google Wiki editing (Ku and Chen, 2015), or reasoning exercises (Soleimanirad and Shangarffam, 2016) to be effective in reducing FLA. Cooperative learning enhances student–student interactions and thus changes the classroom environment, which plays a crucial role in the experience of L2 enjoyment and anxiety, confirming previous research (Dewaele and MacIntyre, 2014). As we found that group work by itself could not reduce anxiety (Yayli, 2017), the combination of cooperative learning with a task outside of course materials is recommended. Another important finding is that a focus on teacher-student interaction enhancement is likely to be effective. Portfolio assessment (Nosratinia and Abdi, 2017) and teacher feedback, feed up, and feed forward methods (Zarrinabadi and Rezazadeh, 2020) that enhanced teacher-student communication could reduce more FLA in comparison with a non-interventional group. These findings confirmed Aida’s (1994) suggestion that L2 teachers had the important role in lessening classroom tension and in creating a friendly, supportive atmosphere that could help reduce FLA. These methods can be included as a complement or as a main task in L2 skill development processes for reducing FLA. They are considered feasible and realistic in L2 educational contexts.

Interaction “involves teachers, learners, and others acting upon each other and consciously or unconsciously interpreting those actions” (Oxford, 1997, p. 444). Foreign language education is characterized by being interactive; however, the interactive FLAR interventions allow learners to communicate with others even more frequently and in meaningful ways. In other words, these interventions can change interactions in L2 education. Many interventions reviewed in this article utilized smaller groups or pairs, which tend to engender a better atmosphere, more individual use of L2, and the creation of closer social bonds with peers (Dewaele and MacIntyre, 2014). We assume that the quantity and quality of oral and written interactions were enhanced by the classroom environment (i.e., a nonthreatening atmosphere and pair/group work) created by the teacher/researcher conducting these interventions. Learners with high FLA tend to distrust social situations in which they must interact in L2; however, anxious learners in the successful interventions could begin to trust the social situations requiring use of L2 through meaningful interactions with teachers, peers, and others. Their FLA level lowered because the fear of being laughed at, embarrassed, or misunderstood, which are considered to be social causes of FLA (MacIntyre, 2017), had been removed through frequent and meaningful interactions. These findings are in agreement with previous suggestions that creating a nonthreatening classroom and community through a friendly climate (Lucas, 1984; Crookall and Oxford, 1991) or focusing on authentic communication (Phillips, 1998) are effective in reducing FLA.

Furthermore, the present review found controversial evidence concerning the effectiveness of CMC on FLA. Despite similarity among studies (e.g., tasks using virtual world), the results of the intervention studies on FLA were inconsistent. Use of a virtual world system called *Second Life* (Melchor-Couto, 2017) resulted in a significant FLA decrease after the intervention while the same system used in Yu et al. (2020) did not. Another virtual world system called *Yoowalk* for grammar practice in speaking (Kruk, 2016) also did not reduce FLA. Results of text chat intervention were also inconsistent. Use of text chat in Arnold (2007) successfully reduced FLA, while synchronous text chat in Baralt and Gurzynski-Weiss (2011) did not. Further experimental research is necessary to confirm the effectiveness of various types of CMC.

Finally, this review has highlighted a need for further research for interventions including performance such as drama. This type of interventions has a high potential of bringing positive feelings at the individual level and a positive atmosphere to classrooms and thus need to be studied with a sufficient number of participants and a sufficient duration, using appropriate procedures and methods of analyses to examine their effectiveness. Additional research is needed to provide more objective evidence regarding what works, in what situations, and why. Creating a positive atmosphere in classrooms, promoting positive feelings in learners, and helping learners develop their skills to manage feelings and beliefs are valued, and there are indications that some methods are effective.

## Limitations

Our analysis was dependent on information published only in English, so we do not claim that our dataset is fully comprehensive. Moreover, due to the complex nature of FLA interventions, it was not always straightforward to determine the category of a particular method. In spite of the study’s limitations, the tables and figures presented in this article are robust enough to reflect accurate and meaningful tendencies.

## Conclusion

This study provides the first systematic review of various classroom interventions and their influence on FLA. It revealed that experimental FLAR intervention studies have been fueled by the trends of (1) reporting scale reliability, (2) including a control group, (3) improving the length of interventions, (4) having an appropriate number of participants, (5) using mean-based statistical analysis, and (6) reporting both within-group and between-group differences. This information can be useful in developing a new intervention and conducting experimental research.

The review showed that FLAR interventions can affect individual and interactional dimensions. Awareness of these different ways to approach FLAR interventions can assist L2 researchers and teachers in establishing specific goals and strategies for their environment. We hope that this study’s systematic organization of information on FLAR methods can begin to provide answers for L2 learners who need to develop anxiety management skills and to educators who are working to create a low-anxiety learning environment and bring a positive atmosphere to classrooms.

Finally, based on the results of this review, we recommend the cooperative digital storytelling approach by Liu et al. (2018) for L2 educators since this method can (1) enhance both linguistic and nonlinguistic (i.e., psychological and social) skills, (2) stimulate imagination, creativity, enthusiasm, and joy, and (3) change both individual and interactional dimensions in L2 learning, without being too demanding of teachers.

## Author Contributions

MT and YY conducted the systematic review and contributed to the final manuscript.

## Conflict of Interest

The authors declare that the research was conducted in the absence of any commercial or financial relationships that could be construed as a potential conflict of interest.
